# Immune-related adverse events in small-cell lung cancer patients treated with immune checkpoint inhibitors: a comprehensive analysis from the FDA adverse event reporting system

**DOI:** 10.3389/fphar.2024.1398667

**Published:** 2024-10-30

**Authors:** Yifeng Bai, Xiaomei Wang, Xiaoqin Dai, Qinghua Ma, Honglin Hu

**Affiliations:** ^1^ Department of Oncology, Sichuan Provincial People’s Hospital, School of Medicine, University of Electronic Science and Technology of China, Chengdu, China; ^2^ Chinese Academy of Sciences Sichuan Translational Medicine Research Hospital, Chengdu, China; ^3^ Department of Emergency, Sichuan Provincial People’s Hospital, School of Medicine, University of Electronic Science and Technology of China, Chengdu, China; ^4^ Department of Traditional Chinese Medicine, Sichuan Provincial People’s Hospital, School of Medicine, University of Electronic Science and Technology of China, Chengdu, China; ^5^ Department of Nursing, Sichuan Provincial People’s Hospital, School of Medicine, University of Electronic Science and Technology of China, Chengdu, China

**Keywords:** SCLC, FDA adverse event reporting system (FAERS), ICIs, immune check point inhibitors, irAEs (immune-related adverse events), adverse (side) effects

## Abstract

**Background:**

The discovery and development of immune checkpoint inhibitors (ICIs) have resulted in their application as a novel therapeutic strategy for patients with small-cell lung cancer (SCLC). However, a comprehensive analysis of the potential adverse effects of ICIs in patients with SCLC remains to be conducted.

**Methods:**

Adverse event (ADE) reports relating to SCLC patients, submitted to the FDA Adverse Event Reporting System (FAERS) from the first quarter of 2013 to the second quarter of 2022, were extracted for analysis. The extracted data were subsequently screened and analyzed using the reporting odds ratio (ROR) method to assess the AE reports.

**Results:**

A total of 4,522 ADE reports were obtained from patients with SCLC who had received either chemotherapy alone or a combination of ICIs with chemotherapy. The ROR analysis identified a total of 91 immune-related adverse events in SCLC patients associated with the ICIs (SCLC-irAEs).

**Conclusion:**

This study revealed that the adverse effects resulting from irAEs in SCLC patients predominantly affected the hematologic and gastrointestinal systems, with the most severe cases potentially leading to fatality.

## 1 Introduction

Lung cancer is a malignant disease, which poses a serious health problem on global scale, as it has a high mortality and morbidity rate (Sung et al., 2021). Small cell lung cancer (SCLC) is a highly aggressive neuroendocrine tumor that accounts for about 15% of all lung cancers (Siegel et al., 2020), with smoking as the main causative risk factor (Govindan et al., 2006). It is characterized by a good initial response to chemotherapy and radiotherapy, but the high proliferative capacity of these type of cancer cells leads to a high recurrence rate and early metastasis rate (Qin et al., 2020). Platinum-based chemotherapy is the first-line treatment of choice for both limited-stage SCLC (LD-SCLC) and extensive-stage SCLC (ED-SCLC) (Yang et al., 2019; Amarasena et al., 2015). However, the advent of various immune checkpoint inhibitors (ICIs), including programmed cell death protein-1 (PD-1) inhibitors and programmed death ligand-1 (PD-L1) inhibitors, offers a suitable alternative, that has dramatically changed the treatment paradigm of SCLC (Reck et al., 2016).

In patients with SCLC, the initial response to chemotherapy frequently leads to substantial tumor cell death. This cytotoxic process may induce the release of tumor-associated antigens (TAAs), including neoantigens. The release of these antigens may enhance tumor immunogenicity, potentially creating a more conducive microenvironment for immunotherapeutic interventions. This immunological rationale has prompted investigations into the combination of ICIs with chemotherapy as a potentially efficacious treatment modality (Kalemkerian and Schneider, 2017). Emerging clinical studies have demonstrated encouraging outcomes for this combinatorial approach in patients with SCLC (Horn et al., 2018; Rudin et al., 2020). The IMPower133 (Horn et al., 2018) trial evaluated the efficacy of atezolizumab in combination with chemotherapy (carboplatin and etoposide) in patients with ED-SCLC and showed an improved median OS for this group compared to the control group (123 months vs 103 months, HR = 0.70, and 95% CI: 0.54 to 0.91, P = 0,007) and prolonged median PFS (52 months vs. 43 months, HR = 0.77, 95% CI: 0.62 to 0.96, P = 002). Furthermore, the KEYNOTE604 (Rudin et al., 2020) study demonstrated that treatment with a combination of Pembrolizumab combined with etoposide and platinum (EP) was found to significantly prolong median PFS compared with the control group (HR = 0.75, 95% CI: 0.61 to 0.91, P = 00023). However, although the results of immunotherapy are encouraging, resultant adverse effects are becoming gradually apparent, which should be a consideration for clinicians.

While the efficacy of ICIs in SCLC treatment is promising, it is imperative to consider their safety profile. Previous studies have reported a spectrum of immune-related adverse events (irAEs) associated with ICI administration in SCLC patients. For instance, Zhang et al. observed that 57% of SCLC patients treated with ICIs experienced irAEs, with the most prevalent being endocrine system toxicity, cutaneous reactions, and immune-mediated pneumonitis (Zhang et al., 2024). Similarly, Lee et al. reported that 39.9% of SCLC patients receiving ICIs experienced irAEs, compared to 24.5% in the placebo group (Lee et al., 2024). The efficacy of ICIs, which stems from their ability to modulate the immune system to inhibit tumor growth, is counterbalanced by the substantial burden associated with irAEs, which exhibit considerable heterogeneity in severity and onset (Elia et al., 2020). However, for SCLC, where the implementation of ICIs is relatively recent, the documentation of adverse events is less comprehensive compared to other malignancies. Moreover, the unpredictable nature of irAEs presents substantial diagnostic and management challenges for clinicians, particularly given the rarity and limited documentation of these events in real-world clinical settings. Considering the potential for severe irAEs in SCLC patients receiving ICIs, a comprehensive understanding of these events is essential for optimal patient management and informed treatment decisions. Our study aims to address this knowledge gap by conducting a systematic analysis of irAEs in SCLC patients utilizing data from the FDA Adverse Event Reporting System (FAERS).

In this study, we introduce the term “SCLC-irAEs” to denote irAEs specifically observed in SCLC patients undergoing treatment with ICIs. SCLC-irAEs represent a distinct subset of irAEs that manifest in the context of SCLC treatment with ICIs, administered either as monotherapy or in combination with chemotherapy. SCLC-irAEs have the potential to affect multiple organ systems and may exhibit distinct patterns in terms of presentation or frequency when compared to irAEs observed in other malignancies treated with ICIs.

## 2 Methods

### 2.1 FAERS data collection and pre-processing

The FAERS database contains information on adverse drug events and medication error reports submitted by health professionals, patients, and manufacturers, predominantly but not exclusively based in the United States (Zhou et al., 2023). FAERS data are stored in ASCII and XML formats, with information categorized as: patient demographic information (DEMO), drug information (DRUG), adverse events (REAC), patient outcomes (OUTC), reporting source (RPSR), treatment start date and end date of the reported drug (THER) and indication for dosing (INDI). We extracted records, deposited during the period from Q1 2013 to Q2 2022, relating to SCLC patients where the primary suspect (PS) was an ICI that had been administered in combination with chemotherapy or chemotherapy in isolation (Supplementary Table S1). A total of 5,784 reports met these criteria. As the database is updated quarterly, patient reporting information may change and inevitably result in duplicates of previously published reports, so it was necessary to deduplicate the records by using the published deletion file. According to the FDA recommendations, we removed the ADE reports with the same gender, age, reporting country, time date and adverse drug reactions (Shen et al., 2024; Gu et al., 2023). The FAERS database was coded using the Medical Dictionary for Regulatory Activities (MedDRA) (Brown et al., 1999). We used the preferred terms (PTs) hierarchy in the MedDRA terminology set to define the ADE descriptive terms. Mortality data in this study were obtained from the “outcome” field in the FAERS reports. It is important to note that these reports capture deaths that occurred during or after the reported adverse events; however, they do not provide information on long-term survival outcomes. Our analysis focused on the association between reported SCLC-irAEs and mortality reports, rather than on long-term survival outcomes. Our investigation focused on analyzing the incidence of SCLC-irAEs utilizing data extracted from the FAERS database.

### 2.2 Reporting odds ratio (ROR) calculations

These calculations are used to detect signals of disproportionate reporting of adverse drug reactions in spontaneous reporting systems. The correlation between a target drug (ICIs in combination with chemotherapy) and a specified ADE is assessed by comparing the proportion of specified ADEs documented as being caused by the target drug with the proportion of identical ADEs occurring with other drugs (chemotherapy), i.e., background frequency. After obtaining data (such as the number of ADE reports for the target drug and other drugs) based on the four-compartment table of the proportional imbalance method, ROR values were calculated based on the formulae:

$$\text{ROR}=\frac{ad}{bc},{\text{ROR}}_{025}=e^{\mathrm{Ln}\left(ROR\right)-1.96\sqrt{\frac{1}{a}+\frac{1}{b}+\frac{1}{c}+\frac{1}{d}}},{\text{ROR}}_{975}=e^{\mathrm{Ln}\left(ROR\right)+1.96\sqrt{\frac{1}{a}+\frac{1}{b}+\frac{1}{c}+\frac{1}{d}}}$$

The lower limit of the 95% confidence interval (CI) of ROR and the ADE signals with the number of reports (a) ≥ 3 and ROR_025_ > 1 were screened (Khaleel et al., 2022).

### 2.3 Statistical analysis

We used the Mann-Whitney U test to compare differences in continuous variables between the two groups. We used the Kruskal-Wallis test to compare differences in continuous variables between multiple groups. The ggplot2 R package was used to visualize data in the form of heat maps, box plots and bar charts. Univariate and multifactorial logistic regression analyses were used to explore the effect of each variable on the clinical prognosis of patients. P-values were considered statistically significant with a two-sided p-value less than 0.05. All data analysis and visualization for this study were done in R software.

## 3 Results

### 3.1 SCLC-irAEs detection in FAERS database

**FIGURE 1:**
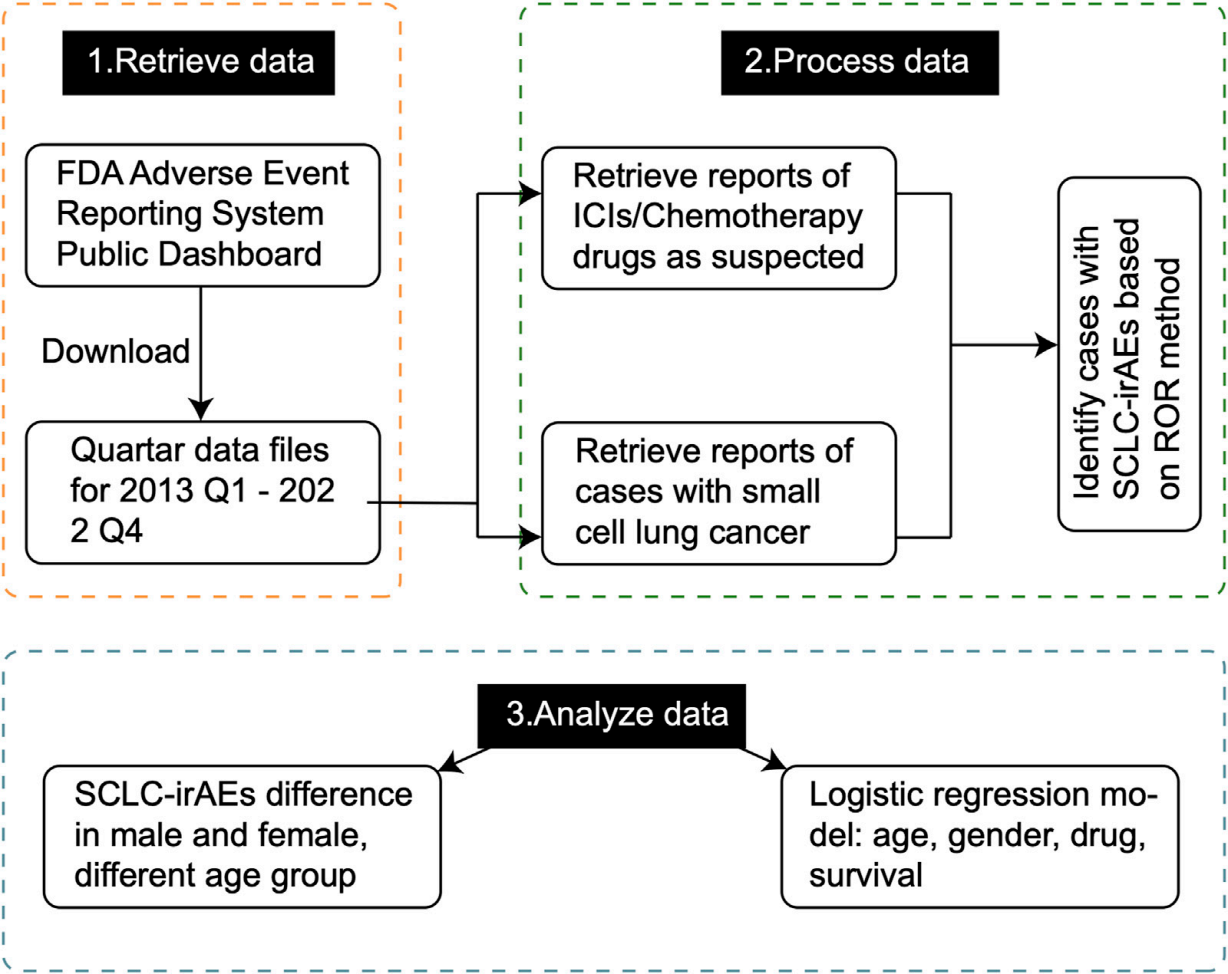
The overall design of the study. This flowchart provides a comprehensive overview of our study methodology, illustrating the step-by-step process from data collection to analysis. It outlines how we extracted and processed data from the FAERS database, and conducted statistical analyses to identify SCLC-irAEs.

**FIGURE 2:**
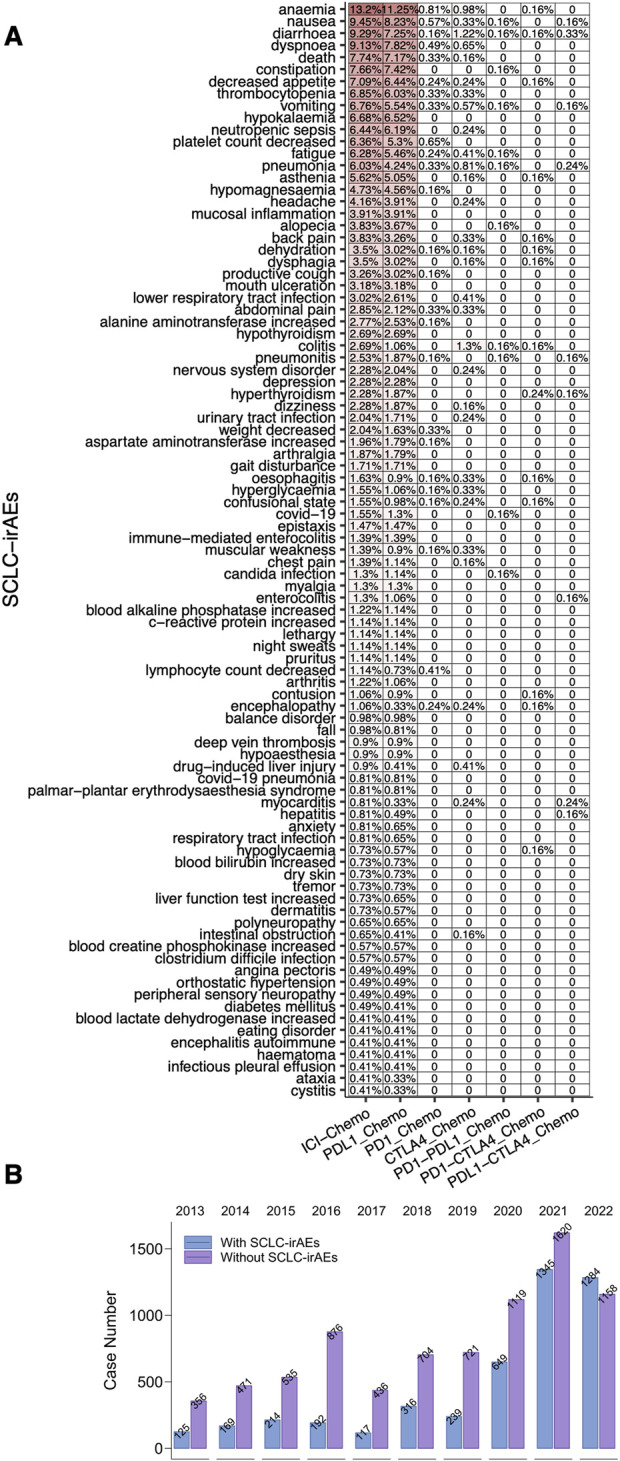
The SCLC-irAEs in the FAERS database. **(A)** A heatmap depicting the frequency of 91 SCLC-irAEs in the FAERS, providing a visual representation of the most common adverse events. **(B)** A bar plot showing the number of SCLC-irAEs cases reported between 2013–2022, illustrating trends over time.

We analyzed the deduplicated sets of reports we extracted (using the criteria detailed in the methodology) from the FAERS database (Figure 1). For the patient group receiving the target drug, we detected 91 associated ADEs, with the top 20 being: anemia, nausea, diarrhea, dyspnoea, death, constipation, decreased appetite, thrombocytopenia, vomiting, hypokalemia, neutropenic sepsis, platelet count decreased, fatigue, pneumonia, asthenia, hypomagnesaemia, headache, mucosal inflammation, alopecia and back pain (a ≥ 3, ROR025 > 1, Figure 2A; Supplementary Table S2). We compared the number of reports of SCLC-irAEs for patient in the period 2013–2022 (Figure 2B).

### 3.2 Correlation between SCLC-irAEs and clinical characteristic

**FIGURE 3:**
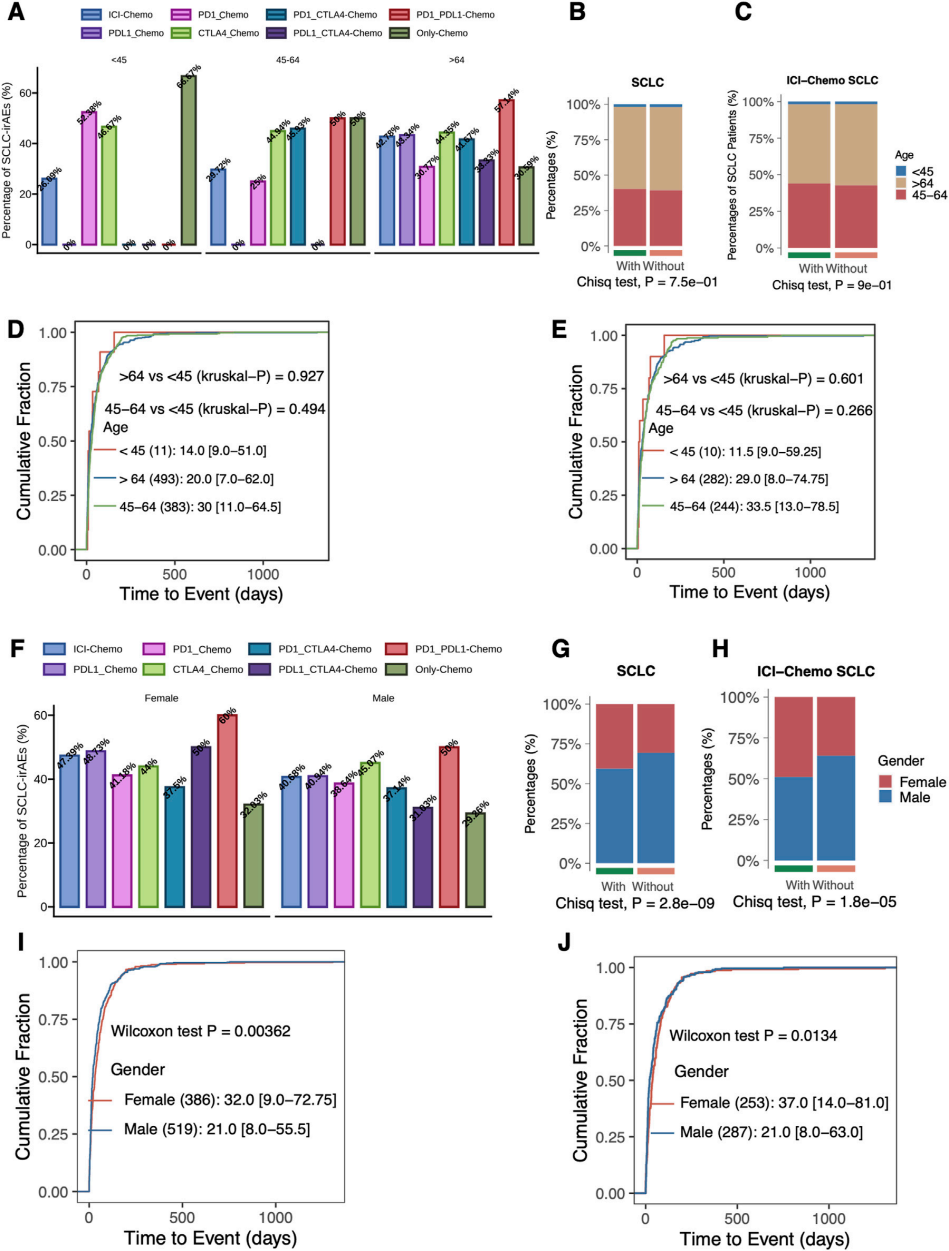
Evaluation of the association between SCLC-irAEs and clinical characteristics. This figure explores the relationships between SCLC-irAEs and patient demographics: **(A)** A bar plot showing the percentage of SCLC-irAEs among different treatment regimens and age groups, illustrating how age may influence adverse event rates. **(B)** A comparison of age group distributions between patients with and without SCLC-irAEs, highlighting any age-related trends in adverse event occurrence. **(C)** A similar age group comparison, but specifically for patients treated with ICI_Chemo, focusing on this important treatment regimen. **(D)** A cumulative distribution function of time-to-event for SCLC-irAEs, grouped by age, showing how quickly adverse events develop in different age groups. **(E)** A similar time-to-event analysis, but specifically for patients treated with ICI_Chemo. **(F)** A bar plot depicting SCLC-irAEs percentages by treatment regimen and gender, illustrating any gender-specific trends. **(G)** A comparison of gender distributions between patients with and without SCLC-irAEs, highlighting any gender-related trends in adverse event occurrence. **(H)** A similar gender comparison, but specifically for patients treated with ICI_Chemo. **(I)** A cumulative distribution function of time-to-event for SCLC-irAEs, grouped by gender, showing how quickly adverse events develop in males versus females. **(J)** A similar time-to-event analysis by gender, but specifically for patients treated with ICI_Chemo.

The clinical characteristics of SCLC patients were detailed in the Supplementary Table S3. We compared the proportion of SCLC-irAEs in patients of different ages (<45, 45–64, >64) under different treatment regimens (Figure 3A). When comparing the with and without SCLC-irAEs groups, in terms of age (Figure 3B), we found no significant differences. In contrast, among SCLC patients using target drugs (ICIs + Chemo), there was a significant difference between the with and without SCLC-irAEs groups in terms of age (*p* < 0.05, Figure 3C). There was no significant difference in the time from drug administration to the onset of SCLC-irAEs between the different age groups in SCLC patients receiving any type of treatment (ICIs + Chemo or Only Chemo) compared with those receiving target drugs (ICIs + Chemo) (Figures 3D, E). We compared the proportion of SCLC-irAEs by gender under different treatment regimens (Figure 3F). The proportion of women was significantly higher than men in the with SCLC-irAEs group (Figures 3G, H), both in the all drugs (ICIs + Chemo or Only Chemo) group and in the group that had been treated with the target drugs (ICIs + Chemo). Additionally, the time from drug administration to SCLC-irAEs was shorter in men than in women, both in SCLC patients using all drugs (ICIs + Chemo or Only Chemo) and in those using the target drug (ICIs + Chemo) (P < 0.05, Figures 3I, J).

### 3.3 Correlation between SCLC-irAEs and prognosis

**FIGURE 4:**
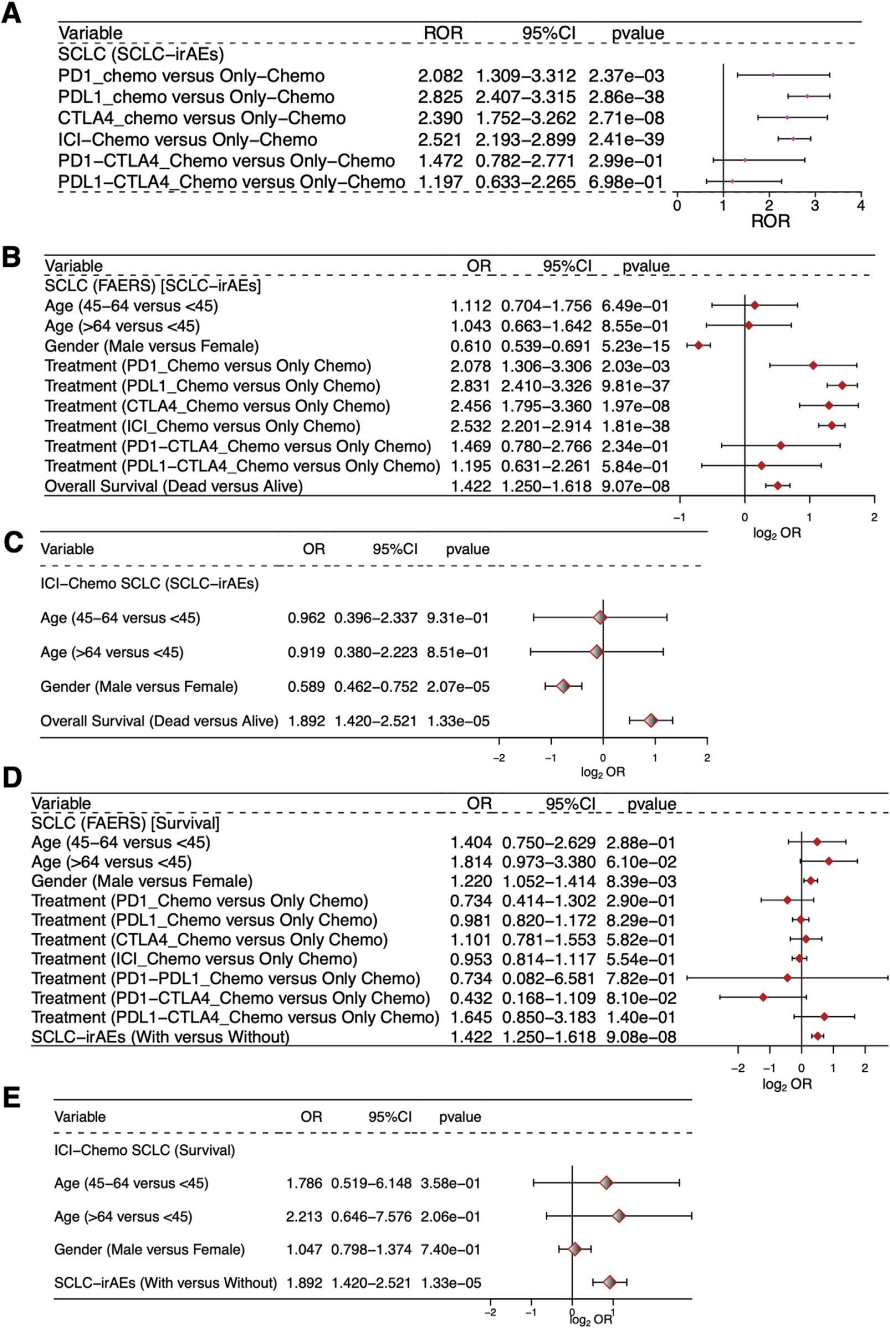
Evaluation of the association between SCLC-irAEs and prognosis. This figure presents statistical analyses of the relationships between SCLC-irAEs, treatment regimens, and patient outcomes: **(A)** A forest plot showing the Reporting Odds Ratio (ROR) and 95% CI for different ICI_Chemo treatment regimens, illustrating the relative risk of adverse events for each treatment. **(B)** A univariable logistic regression model examining the associations between clinical characteristics, prognosis, and SCLC-irAEs for the SCLC patients in the study. **(C)** A univariable logistic regression model examining the associations between clinical characteristics, prognosis, and SCLC-irAEs for the SCLC patients treated with ICI_Chemo. **(D)** A univariable logistic regression model exploring how clinical characteristics and SCLC-irAEs are associated with prognosis for the SCLC patients. **(E)** A univariable logistic regression model exploring how clinical characteristics and SCLC-irAEs are associated with prognosis for the SCLC patients treated with ICI_Chemo.

We used forest plots to show the ROR values and 95% confidence intervals for the occurrence of SCLC-irAEs under different ICIs + Chemo regimens (Figure 4A), such as PD1_Chemo, PDL1_Chemo, CTLA4_Chemo, PD1-CTLA4_Chemo and PDL1-CTLA4_Chemo. Univariable logistic regression analysis showed that death status, PD1_Chemo, PDL1_Chemo, CTLA4_Chemo, and ICI_Chemo were all more likely to occur in SCLC-irAEs within the all drugs (ICIs + Chemo or Only Chemo) group (OR > 1, P < 0.05, Figure 4B). Among SCLC patients who had received the target drugs (ICIs + Chemo), those in the death state were more likely to develop SCLC-irAEs (OR > 1, P < 0.05, Figure 4C). Patients with SCLC-irAEs were prognostically at risk regardless of whether all drugs (ICIs + Chemo or Only Chemo) or target drugs (ICIs + Chemo) were used (OR > 1, P < 0.05, Figures 4D, E).

## 4 Discussion

The immune system plays a crucial role in the treatment of cancer. ICIs, which modulate immune checkpoints, have demonstrated significant improvement in the survival of cancer patients, resulting in their increasing use in clinical practice. Our comprehensive analysis of the FAERS database revealed several key irAEs in SCLC patients treated with ICIs in combination with chemotherapy. The most frequently observed irAEs in our analysis included hematological abnormalities (anemia, thrombocytopenia), gastrointestinal disturbances (nausea, diarrhea, constipation, vomiting), respiratory complications (dyspnea, pneumonia), metabolic imbalances (hypokalemia, hypomagnesemia), and constitutional symptoms (fatigue, asthenia), among other adverse events. Furthermore, we identified severe adverse events, including neutropenic sepsis and treatment-related mortality. These findings exhibit substantial concordance with existing literature on irAEs in SCLC, although with some discernible variations in frequency and severity. The clinical implications of these results are multifaceted and extensive. First, our findings emphasize the critical importance of vigilant monitoring and proactive management of irAEs in SCLC patients receiving ICI-based combination therapy. By elucidating the spectrum and prevalence of these adverse events, clinicians can develop more targeted screening protocols and implement early intervention strategies, potentially reducing the severity of irAEs and improving overall patient outcomes. Additionally, this comprehensive profile of irAEs can guide the development of tailored patient education programs, enabling SCLC patients to recognize and report symptoms promptly, thus facilitating timely medical intervention.

Studies have shown that hematologic irAEs account for 0.6% to 3.6% of all irAEs (Michot et al., 2019; Zheng et al., 2023; Kramer et al., 2021), with the initial irAE occurring, on average (median), within the first 10 weeks of ICIs use (although they have been recorded as commencing as late as over 1 year after the start of treatment), with remission occurring after ∼1 to 2 months of treatment (Michot et al., 2019; Delanoy et al., 2019; Zhou H. et al., 2021). Hematologic irAEs commonly present (9,80%) as anemia, whereas the incidence of thrombocytopenia appears to be rarer (0.94%) (Petrelli et al., 2018). In our investigation, anemia emerged as the predominant hematologic irAE, corroborating the findings of Zheng et al., who reported an incidence of 11.20% in SCLC patients undergoing ICI treatment (Zheng et al., 2023). However, our analysis revealed a higher incidence of thrombocytopenia compared to previous case reports (Zhou H. et al., 2021), indicating potential disparities between controlled clinical trials and real-world settings. In a retrospective study including 20,128 patients, 82 deaths were deemed to be caused by irAEs. Hematologic causes ranked fourth, behind respiratory failure, cardiovascular events, and infections (Zhou X. et al., 2021). The second highest incidence of hematologic irAEs was immune thrombocytopenia (ITP), although it does vary dramatically by geography One study (57 patients) determined i) patient origin as predominantly from North America (53%) followed by and Asia (33%) then Europe (14%); ii) the underlying diseases remained most common in melanoma and NSCLC; iii) 65% were treated with anti-PD-1 monoclonal antibody alone (Davis et al., 2019), 16% with anti-CTLA-4 monoclonal antibody alone, 18% with a combination of both, and 2% with anti-PD-L1 monoclonal antibody alone (Calvo, 2019). The presence of immune disease prior to administration of ICIs may increase the incidence of ITP. Patients may experience varying degrees of skin and mucosal bleeding, and in severe cases, spontaneous visceral bleeding which can even be life-threatening.

With respect to gastrointestinal irAEs, our findings of prevalent nausea, diarrhea, and vomiting are consistent with the observations of Yao et al. (Yao et al., 2024). Notably, despite pneumonitis being frequently cited as a significant concern in ICI therapy, our analysis demonstrated an incidence comparable to that reported in several previous studies. Studies have shown that gastrointestinal (GI) toxicity is very common in irAEs (with lower GI toxicity being more common than upper GI toxicity) often manifesting as diarrhea, colitis, nausea, and vomiting (Rajha et al., 2020), usually 6 to 8 weeks after starting treatment (Weber et al., 2015a). For cancer patients treated with CTLA-4 antibodies, GI toxicity is the most common cause of discontinuation (Reddy et al., 2018). Mild diarrhea or enteritis can be improved by antidiarrheal medications, such as loperamide or atropine, but infection needs to be excluded before use (Naidoo et al., 2015). For severe diarrhea or enterocolitis, oral or intravenous glucocorticoids are the first-line treatment, with 4% to 60% of patients suffering from ICI-associated enterocolitis gaining relief from symptoms though receiving hormones. Infliximab, vedolizumab may be effective in hormone-refractory enterocolitis (Thompson et al., 2020).

The overall incidence of ICIs-related pneumonitis (CIP) is low compared to irAEs of other systems such as gastrointestinal, cutaneous, endocrine, hepatic and renal. In addition, the incidence of CIP varies by tumor type; therapy (mono or combination), additional treatment, and presence or absence of additional underlying pulmonary disease. Currently, the overall incidence of CIP ranges from 3% to 5% (0.8% to 1.0% for severe CIP) (Suresh et al., 2018a; Zhai et al., 2020; Andruska et al., 2018; Inthasot et al., 2020). The incidence of CIP in clinical trials is around 5% (Ribas et al., 2015; Borghaei et al., 2015; Weber et al., 2015b; Herbst et al., 2016; Robert et al., 2014; Robert et al., 2015), and the incidence of CIP in the real world is higher compared to clinical trials, with data suggesting up to 19% (Suresh et al., 2018b). For instance, Zou et al. reported a CIP incidence rate of 6% in a retrospective comparative cohort study (Zou et al., 2023). The comparatively lower rate observed in our study may be attributed to variations in reporting practices or advancements in management strategies implemented in recent years. Studies have shown that about 0.45% of deaths in tumor patients are due to immune-related adverse events, of which immune-related pneumonia accounts for about 28% of total deaths (Wang et al., 2019). The exact process of CIP occurrence is yet to be confirmed, but the following have been reported: 1) an imbalance between effector T cells and Treg in the interstitial lung leading to an inflammatory response (Keir et al., 2008); 2) high activation of alveolar macrophages (Nguyen and Ohashi, 2015); 3) release of cytokines such as interleukin 17 (IL-17) and IL-35, which can lead to pulmonary fibrosis and acute lung injury in addition to tumor immune escape and induction of T-cell dysfunction by suppressing the immune microenvironment (Nicholl et al., 2014; Tang et al., 2018; Zhang et al., 2016); and 4) antibody production involving B cells (Day and Hansen, 2016).

We acknowledge that our study has several limitations stemming from its reliance on the FAERS database. The FAERS data may be subject to reporting bias, as adverse events are more frequently reported for novel therapies or those under heightened surveillance. Additionally, our study lacked a comparison group of patients treated with ICIs who did not experience irAEs. This limitation precludes a comprehensive assessment of the prognostic value of SCLC-irAEs in ICI treatment efficacy. Previous research has suggested that the occurrence of irAEs may serve as a favorable prognostic factor for ICI treatment efficacy, a finding that necessitates further investigation in the context of SCLC. The interpretation of mortality data in this pharmacovigilance study is subject to notable limitations. The absence of long-term follow-up data beyond the point of adverse event reporting impedes the ability to draw definitive conclusions regarding the relationship between SCLC-irAEs and patient mortality. The reported fatalities may be attributed to multiple factors, including disease progression, and require cautious interpretation. Furthermore, the database may contain incomplete or inconsistent information owing to its voluntary reporting nature. To mitigate these limitations and bolster the robustness of our findings, future research should consider several approaches. Integration of multiple data sources, including electronic health records, clinical trial data, and other pharmacovigilance databases, could yield a more comprehensive understanding of adverse events in SCLC patients treated with immune checkpoint inhibitors. The implementation of prospective cohort studies would facilitate more controlled data collection and enhanced assessment of causality between treatments and adverse events. Leveraging data from large-scale, population-based registries could contribute to establishing more accurate incidence rates and enable more robust comparisons between various treatment regimens. Collaborative efforts with healthcare providers to collect and analyze real-world data could yield valuable insights into the clinical management of immune-related adverse events in SCLC patients. By addressing these limitations in future investigations, we aim to furnish a more comprehensive and precise analysis of immune-related adverse events in SCLC patients treated with immune checkpoint inhibitors, ultimately contributing to enhanced patient care and safety.

## 5 Conclusion

In this study, we analyzed SCLC-irAEs based on the FAERS database to extract records relating to SCLC patients that had received either ICIs combined with chemotherapy or chemotherapy-only regimens. Results showed that SCLC-irAEs cause more adverse effects in hematologic, gastrointestinal and systemic diseases, and even death in severe cases. This study provides guidance for the safe clinical use of ICIs in combination with chemotherapy in SCLC patients.

## Data Availability

The original contributions presented in the study are included in the article/Supplementary Material, further inquiries can be directed to the corresponding authors.

## Supplementary material

SUPPLEMENTARY TABLE S1 The drug list of the ICIs and chemotherapy.

SUPPLEMENTARY TABLE S2 The SCLC-irAEs RORs across FAERS SCLC.
