# Supplementary material for: Immune-related adverse events in small-cell lung cancer patients treated with immune checkpoint inhibitors: a comprehensive analysis from the FDA adverse event reporting system
Source: Front Pharmacol. 2024 Oct 30;15:1398667. doi: 10.3389/fphar.2024.1398667 (PMC11558040; doi:10.3389/fphar.2024.1398667)
Supplement: Supplementary file 1 [file Table1.pdf]

Drug_name	Type
Pembrolizumab	immunotherapy
Atezolizumab	immunotherapy
Avelumab	immunotherapy
Durvalumab	immunotherapy
Nivolumab	immunotherapy
Ipilimumab	immunotherapy
Tremelimumab	immunotherapy
Sintilimab	immunotherapy
Tiragolumab	immunotherapy
Epirubicin	chemotherapy
doxorubicin	chemotherapy
Adriamycin	chemotherapy
bleomycin	chemotherapy
Mitomycin	chemotherapy
Daunorubicin	chemotherapy
methotrexate	chemotherapy
Fluorouracil	chemotherapy
Pemetrexed	chemotherapy
Gemcitabine	chemotherapy
irinotecan	chemotherapy
Etoposide	chemotherapy
topotecan	chemotherapy
Teniposide	chemotherapy
lurbinectedin	chemotherapy
bendamustine	chemotherapy
Temozolomide	chemotherapy
Oxaliplatin	chemotherapy
Cisplatin	chemotherapy
Nedaplatin	chemotherapy
carboplatin	chemotherapy
Lobaplatin	chemotherapy
Ifosfamide	chemotherapy
Cyclophosphamide	chemotherapy
Dacarbazine	chemotherapy
Lomustine	chemotherapy

Vinblastine	chemotherapy
vinorelbine	chemotherapy
vincristine	chemotherapy
vindesine sulfate	chemotherapy
Docetaxel	chemotherapy
paclitaxel	chemotherapy
