# Supplementary material for: Immune-related adverse events in small-cell lung cancer patients treated with immune checkpoint inhibitors: a comprehensive analysis from the FDA adverse event reporting system
Source: Front Pharmacol. 2024 Oct 30;15:1398667. doi: 10.3389/fphar.2024.1398667 (PMC11558040; doi:10.3389/fphar.2024.1398667)
Supplement: Supplementary file 2 [file Table2.pdf]

SCLC_ROR	SCLC_P	index	ROR025	SCLC_ici_chemo	SCLC_ac_abcd	SCLC_ac	SCLC_a	reaction	drug
2.2956071	0.000804	abdominal pain[ICI-Chemo]	1.419781	0.028524857	0.01770833	68	35	abdominal pain	ICI-Chemo
4.6258382	8.82E-08	alanine aminotransferase increased[ICI-Chemo]	2.543434	0.027709861	0.01302083	50	34	alanine aminotransferase increased	ICI-Chemo
4.9162228	8.02E-11	alopecia[ICI-Chemo]	2.925617	0.038304808	0.01770833	68	47	alopecia	ICI-Chemo
2.4122126	3.69E-14	anaemia[ICI-Chemo]	1.913406	0.13202934	0.08255208	317	162	anaemia	ICI-Chemo
4.2751843	0.034371	angina pectoris[ICI-Chemo]	1.067435	0.004889976	0.00234375	9	6	angina pectoris	ICI-Chemo
4.2859491	0.009056	anxiety[ICI-Chemo]	1.461804	0.008149959	0.00390625	15	10	anxiety	ICI-Chemo
4.5187255	1.72E-05	arthralgia[ICI-Chemo]	2.195673	0.018744906	0.00885417	34	23	arthralgia	ICI-Chemo
32.326733	4.57E-07	arthritis[ICI-Chemo]	4.265275	0.012224939	0.00416667	16	15	arthritis	ICI-Chemo
3.2381546	0.000259	aspartate aminotransferase increased[ICI-Chemo]	1.713855	0.019559902	0.01041667	40	24	aspartate aminotransferase increased	ICI-Chemo

4.0377011	5.41E-13	asthenia[ICI-Chemo]	2.701247	0.056234719	0.02786458	107	69	asthenia	ICI-Chemo
10.687398	0.014597	ataxia[ICI-Chemo]	1.247271	0.00407498	0.0015625	6	5	ataxia	ICI-Chemo
6.0823529	1.89E-12	back pain[ICI-Chemo]	3.477909	0.038304808	0.01666667	64	47	back pain	ICI-Chemo
8.5925926	0.000176	balance disorder[ICI-Chemo]	2.420367	0.009779951	0.00390625	15	12	balance disorder	ICI-Chemo
5.3774752	0.000257	blood alkaline phosphatase increased[ICI-Chemo]	2.081352	0.012224939	0.00546875	21	15	blood alkaline phosphatase increased	ICI-Chemo
3.2105911	0.026176	blood bilirubin increased[ICI-Chemo]	1.140225	0.007334963	0.00390625	15	9	blood bilirubin increased	ICI-Chemo
7.4905738	0.006314	blood creatine phosphokinase increased[ICI-Chemo]	1.553808	0.005704971	0.00234375	9	7	blood creatine phosphokinase increased	ICI-Chemo

5.341653	0.037662	blood lactate dehydrogenase increased[ICI-Chemo]	1.0349	0.00407498	0.00182292	7	5	blood lactate dehydrogenase increased	ICI-Chemo
3.758244	0.003008	c-reactive protein increased[ICI-Chemo]	1.572431	0.011409943	0.00572917	22	14	c-reactive protein increased	ICI-Chemo
17.248555	7.84E-07	candida infection[ICI-Chemo]	3.959734	0.013039935	0.0046875	18	16	candida infection	ICI-Chemo
2.6082054	0.010761	chest pain[ICI-Chemo]	1.281487	0.013854931	0.00807292	31	17	chest pain	ICI-Chemo
4.9918033	0.015256	clostridium difficile infection[ICI-Chemo]	1.288639	0.005704971	0.00260417	10	7	clostridium difficile infection	ICI-Chemo
4.4860239	2.07E-07	colitis[ICI-Chemo]	2.459533	0.026894866	0.01276042	49	33	colitis	ICI-Chemo
2.2675221	0.018	confusional state[ICI-Chemo]	1.185797	0.015484923	0.00963542	37	19	confusional state	ICI-Chemo
4.007394	4.77E-17	constipation[ICI-Chemo]	2.841362	0.076609617	0.03828125	147	94	constipation	ICI-Chemo
27.970346	3.42E-06	contusion[ICI-Chemo]	3.65485	0.010594947	0.00364583	14	13	contusion	ICI-Chemo

5.1215853	4.33E-05	covid-19[ICI-Chemo]	2.235797	0.015484923	0.00703125	27	19	covid-19	ICI-Chemo
21.462613	8.46E-05	covid-19 pneumonia[ICI-Chemo]	2.744392	0.008149959	0.00286458	11	10	covid-19 pneumonia	ICI-Chemo
10.687398	0.014597	cystitis[ICI-Chemo]	1.247271	0.00407498	0.0015625	6	5	cystitis	ICI-Chemo
2.1537193	1.99E-07	death[ICI-Chemo]	1.610285	0.077424613	0.05026042	193	95	death	ICI-Chemo
3.1927524	1.80E-12	decreased appetite[ICI-Chemo]	2.28465	0.070904645	0.03854167	148	87	decreased appetite	ICI-Chemo
3.3677162	0.016134	deep vein thrombosis[ICI-Chemo]	1.30234	0.008964955	0.0046875	18	11	deep vein thrombosis	ICI-Chemo
2.6750483	1.62E-05	dehydration[ICI-Chemo]	1.703177	0.035044825	0.0203125	78	43	dehydration	ICI-Chemo
20.316931	1.01E-11	depression[ICI-Chemo]	6.164632	0.022819886	0.00807292	31	28	depression	ICI-Chemo
3.8541872	0.017351	dermatitis[ICI-Chemo]	1.288953	0.007334963	0.00364583	14	9	dermatitis	ICI-Chemo
6.4152334	0.015666	diabetes mellitus[ICI-Chemo]	1.292954	0.004889976	0.00208333	8	6	diabetes mellitus	ICI-Chemo
2.5214841	2.37E-11	diarrhoea[ICI-Chemo]	1.912768	0.092909535	0.05625	216	114	diarrhoea	ICI-Chemo
3.7904504	1.24E-05	dizziness[ICI-Chemo]	2.043059	0.022819886	0.01145833	44	28	dizziness	ICI-Chemo

5.9002878	0.001193	drug-induced liver injury[ICI-Chemo]	1.874997	0.008964955	0.00390625	15	11	drug-induced liver injury	ICI-Chemo
3.2105911	0.026176	dry skin[ICI-Chemo]	1.140225	0.007334963	0.00390625	15	9	dry skin	ICI-Chemo
5.2357827	1.91E-10	dysphagia[ICI-Chemo]	3.007207	0.035044825	0.01588542	61	43	dysphagia	ICI-Chemo
2.7218284	1.43E-12	dyspnoea[ICI-Chemo]	2.049196	0.091279544	0.05338542	205	112	dyspnoea	ICI-Chemo
5.341653	0.037662	eating disorder[ICI-Chemo]	1.0349	0.00407498	0.00182292	7	5	eating disorder	ICI-Chemo
10.687398	0.014597	encephalitis autoimmune[ICI-Chemo]	1.247271	0.00407498	0.0015625	6	5	encephalitis autoimmune	ICI-Chemo
3.0982976	0.01213	encephalopathy[ICI-Chemo]	1.32079	0.010594947	0.00572917	22	13	encephalopathy	ICI-Chemo
4.9187212	0.000256	enterocolitis[ICI-Chemo]	2.018294	0.013039935	0.00598958	23	16	enterocolitis	ICI-Chemo
6.4689826	1.58E-05	epistaxis[ICI-Chemo]	2.56144	0.014669927	0.00625	24	18	epistaxis	ICI-Chemo
3.6768959	0.007414	fall[ICI-Chemo]	1.444029	0.009779951	0.00494792	19	12	fall	ICI-Chemo
3.0024714	2.13E-10	fatigue[ICI-Chemo]	2.116936	0.062754686	0.03489583	134	77	fatigue	ICI-Chemo

15.149254	1.75E-08	gait disturbance[ICI-Chemo]	4.510066	0.017114914	0.00625	24	21	gait disturbance	ICI-Chemo
10.687398	0.014597	haematoma[ICI-Chemo]	1.247271	0.00407498	0.0015625	6	5	haematoma	ICI-Chemo
4.1536281	3.50E-10	headache[ICI-Chemo]	2.592029	0.041564792	0.0203125	78	51	headache	ICI-Chemo
3.0590445	0.033957	hepatitis[ICI-Chemo]	1.161634	0.008149959	0.00442708	17	10	hepatitis	ICI-Chemo
6.8340232	6.09E-06	hyperglycaemia[ICI-Chemo]	2.722484	0.015484923	0.00651042	25	19	hyperglycaemia	ICI-Chemo
12.180817	2.05E-10	hyperthyroidism[ICI-Chemo]	4.691899	0.022819886	0.00859375	33	28	hyperthyroidism	ICI-Chemo
2.9456209	0.028931	hypoaesthesia[ICI-Chemo]	1.181827	0.008964955	0.00494792	19	11	hypoaesthesia	ICI-Chemo
4.8195813	0.00638	hypoglycaemia[ICI-Chemo]	1.481319	0.007334963	0.00338542	13	9	hypoglycaemia	ICI-Chemo
6.1661135	5.38E-21	hypokalaemia[ICI-Chemo]	4.035433	0.066829666	0.02916667	112	82	hypokalaemia	ICI-Chemo
7.1528372	1.54E-16	hypomagnesaemia[ICI-Chemo]	4.196309	0.047269764	0.01979167	76	58	hypomagnesaemia	ICI-Chemo
4.7869347	8.99E-08	hypothyroidism[ICI-Chemo]	2.59017	0.026894866	0.0125	48	33	hypothyroidism	ICI-Chemo

4.0650138	0.000546	immune-mediated enterocolitis[ICI-Chemo]	1.806852	0.013854931	0.00677083	26	17	immune-mediated enterocolitis	ICI-Chemo
10.687398	0.014597	infectious pleural effusion[ICI-Chemo]	1.247271	0.00407498	0.0015625	6	5	infectious pleural effusion	ICI-Chemo
3.4231337	0.033298	intestinal obstruction[ICI-Chemo]	1.117549	0.006519967	0.00338542	13	8	intestinal obstruction	ICI-Chemo
10.04122	2.60E-05	lethargy[ICI-Chemo]	2.880296	0.011409943	0.00442708	17	14	lethargy	ICI-Chemo
9.6465517	0.000955	liver function test increased[ICI-Chemo]	2.081144	0.007334963	0.00286458	11	9	liver function test increased	ICI-Chemo
16.217815	1.60E-14	lower respiratory tract infection[ICI-Chemo]	6.358076	0.030154849	0.0109375	42	37	lower respiratory tract infection	ICI-Chemo
15.067601	6.60E-06	lymphocyte count decreased[ICI-Chemo]	3.419139	0.011409943	0.00416667	16	14	lymphocyte count decreased	ICI-Chemo

85.747475	1.84E-18	mouth ulceration[ICI-Chemo]	11.76689	0.031784841	0.01041667	40	39	mouth ulceration	ICI-Chemo
35.419847	3.97E-21	mucosal inflammation[ICI-Chemo]	11.01034	0.039119804	0.01328125	51	48	mucosal inflammation	ICI-Chemo
2.2804236	0.025554	muscular weakness[ICI-Chemo]	1.148292	0.013854931	0.00859375	33	17	muscular weakness	ICI-Chemo
3.1252909	0.00442	myalgia[ICI-Chemo]	1.446052	0.013039935	0.00703125	27	16	myalgia	ICI-Chemo
7.1487264	0.001117	myocarditis[ICI-Chemo]	1.963906	0.008149959	0.00338542	13	10	myocarditis	ICI-Chemo
2.0270308	1.01E-07	nausea[ICI-Chemo]	1.561353	0.094539527	0.06354167	244	116	nausea	ICI-Chemo
20.316931	1.01E-11	nervous system disorder[ICI-Chemo]	6.164632	0.022819886	0.00807292	31	28	nervous system disorder	ICI-Chemo
6.1316833	3.31E-20	neutropenic sepsis[ICI-Chemo]	3.98398	0.064384678	0.028125	108	79	neutropenic sepsis	ICI-Chemo
30.146744	1.16E-06	night sweats[ICI-Chemo]	3.959742	0.011409943	0.00390625	15	14	night sweats	ICI-Chemo
2.5303377	0.00653	oesophagitis[ICI-Chemo]	1.320745	0.016299919	0.00963542	37	20	oesophagitis	ICI-Chemo

6.4152334	0.015666	orthostatic hypertension[ICI-Chemo]	1.292954	0.004889976	0.00208333	8	6	orthostatic hypertension	ICI-Chemo
10.727198	0.000362	palmar-plantar erythrodysaesthesia syndrome[ICI-Chemo]	2.346858	0.008149959	0.003125	12	10	palmar-plantar erythrodysaesthesia syndrome	ICI-Chemo
4.2751843	0.034371	peripheral sensory neuropathy[ICI-Chemo]	1.067435	0.004889976	0.00234375	9	6	peripheral sensory neuropathy	ICI-Chemo
2.7477309	2.36E-09	platelet count decreased[ICI-Chemo]	1.957266	0.063569682	0.03671875	141	78	platelet count decreased	ICI-Chemo
1.4886287	0.012443	pneumonia[ICI-Chemo]	1.098528	0.060309698	0.04739583	182	74	pneumonia	ICI-Chemo
1.7564029	0.027661	pneumonitis[ICI-Chemo]	1.087612	0.025264874	0.01796875	69	31	pneumonitis	ICI-Chemo
4.2805578	0.023955	polyneuropathy[ICI-Chemo]	1.286507	0.006519967	0.003125	12	8	polyneuropathy	ICI-Chemo
17.577085	6.56E-16	productive cough[ICI-Chemo]	6.919713	0.032599837	0.01171875	45	40	productive cough	ICI-Chemo
2.3083265	0.043575	pruritus[ICI-Chemo]	1.081711	0.011409943	0.00703125	27	14	pruritus	ICI-Chemo

7.1487264	0.001117	respiratory tract infection[ICI-Chemo]	1.963906	0.008149959	0.00338542	13	10	respiratory tract infection	ICI-Chemo
1.8278111	8.11E-05	thrombocytopenia[ICI-Chemo]	1.357124	0.068459658	0.04817708	185	84	thrombocytopenia	ICI-Chemo
3.2105911	0.026176	tremor[ICI-Chemo]	1.140225	0.007334963	0.00390625	15	9	tremor	ICI-Chemo
2.9984748	0.000402	urinary tract infection[ICI-Chemo]	1.629693	0.020374898	0.01119792	43	25	urinary tract infection	ICI-Chemo
2.6749772	1.72E-09	vomiting[ICI-Chemo]	1.929193	0.067644662	0.03958333	152	83	vomiting	ICI-Chemo
3.1760791	0.000227	weight decreased[ICI-Chemo]	1.708689	0.020374898	0.0109375	42	25	weight decreased	ICI-Chemo
