# Supplementary material for: Immune-related adverse events in small-cell lung cancer patients treated with immune checkpoint inhibitors: a comprehensive analysis from the FDA adverse event reporting system
Source: Front Pharmacol. 2024 Oct 30;15:1398667. doi: 10.3389/fphar.2024.1398667 (PMC11558040; doi:10.3389/fphar.2024.1398667)
Supplement: Supplementary file 3 [file Table3.pdf]

Supplementary Table 3. The clinical characteristics of the SCLC patients in this study.

		Overall (N = 3,840)	ICI-Chemo (N = 1,227)	Only_Chemo (N = 2,613)	P-Value
Age (median [IQR])		66.00 [59.00, 72.00]	66.00 [60.00, 72.00]	66.00 [58.00, 72.00]	0.342
Gender (%)	Female	1163 (35.4)	514 (44.0)	649 (30.6)	<0.001
	Male	2123 (64.6)	654 (56.0)	1469 (69.4)	
